# Analysis of Precipitation Characteristics during 1957-2012 in the Semi-Arid Loess Plateau, China

**DOI:** 10.1371/journal.pone.0141662

**Published:** 2015-11-03

**Authors:** Weijun Zhao, Xinyang Yu, Huan Ma, Qingke Zhu, Yan Zhang, Wei Qin, Ning Ai, Yu Wang

**Affiliations:** 1 Key Laboratory of tourism and resources environment in colleges and universities of Shandong Province, Taishan University, Taian, Shandong, China; 2 Beijing Forestry University, Forestry Ecological Engineering Research Center, Ministry of Education, Beijing, China; 3 College of Resources and Environment, Shandong Agricultural University, Taian, China; 4 Department of Sedimentation, China Institute of Water Resources and Hydropower Research, Beijing, 100044, China; CNRS, FRANCE

## Abstract

Precipitation is the only water supply and most important factor affecting vegetation growth on the slopes of semi-arid Loess Plateau of China. Based on precipitation data from 7 synoptic stations in the study area over the period 1957–2012, the trends of precipitation and standardized precipitation index (SPI) were analyzed by using linear regression, Mann−Kendall, and Spearman’s Rho tests at the 5% significance level. The results show that (1) the precipitation fluctuation of monthly precipitation was intense (coefficients of variation> 100%), and the drier years were recorded as 1965 and 1995 at all stations. (2) The significant change trend of different stations varied on different time scales: the Changwu station had a significant decreasing trend in April (−0.488 mm/year) and November (−0.249 mm/year), while Luochuan station was in April (−0.457 mm/year); Changwu station displayed a significant increasing trends in winter (0.220 mm/year) and a significant decreasing trends in spring (−0.770 mm/year). The significant decreasing trends in annual precipitation were detected at the Suide (−2.034 mm/year) and Yan’an (–2.129 mm/year) stations. (3) The SPI−12 series analysis suggests that the drought degree of Yulin and Changwu was the lowest and that of Hengshan was the highest among the 7 synoptic stations.

## Introduction

The main source of the soil moisture on the slopes of the semi-arid Loess Plateau is atmospheric precipitation. It exerts a direct impact on the dynamic change in the soil moisture [1–3], especially during droughts [4]. Droughts can be defined as a temporary imbalance of water availability consisting of a persistent lower than average precipitation of uncertain frequency, duration, and severity. Droughts are unpredictable or extremely hard to predict resulting in diminished water availability [5–6]. They can directly impact meteorology, agriculture, hydrology, and socio—economy. The different ways in which droughts have been parameterized is a major hindrance for drought management [7]. Previous studies [8–9] aimed at the stochastic characterization of droughts by applying log-linear modeling and Markovchain modeling, respectively, to drought class transitions derived from SPI time series. In addition, there have been numerous studies on drought and a variety of indices developed for indicating drought [10–14].

Trends in drought occurrence frequency or duration can be explained via changes in precipitation [15]. In recent years, precipitation trends in a number of locations have been compared and analyzed by scientists [10, 16–20]. In Asia, Gemmer et al.[19] analyzed the annual rainfall series of 160 stations in China. They observed a spatial clustering of the trends in certain months, including distinct trend belts in east and northeast China. Over the Indian sub-continent, rainfall analysis between 1871 and 1994 indicated decreasing trends during 1880–1905 and 1945–1965 with increasing trends during other periods [21]. Similar studies of India reveal that there are significant differences in rainfall trends at the regional level [22–23]. Trends in long-term rainfall in Turkey showed significant trends in January, February, and September and in the annual means [18]. Negative trends were found in approximately 60% of the stations studied in Iran with the significant trends occurring in the northwest part of the country [16].

Furthermore, there have been a plethora of precipitation studies and reports for different periods and locations in China, especially in the semi−arid Loess Plateau. Liu et al. [20] investigated spatial and temporal variability of annual precipitation during 1961–2006 in the Yellow River Basin. Wang and Zhang [14] analyzed drought-flood spatial-temporal characteristics in the eastern region of Gansu during the past 40 years, based on a standard precipitation index. Precipitation trends have also been studied in the south and northeast of China to provide information on climate variability [24–25]. Meanwhile, some researchers have analyzed the dynamic characteristics of monthly, seasonal, and annual precipitations in the semi-arid Loess Plateau [26–27]. Fu and Wang [27] analyzed the change of monthly precipitation at 51 stations for the period 1961–2000 by empirical orthogonal function and wavelet analysis. While, the spatiotemporal change of seasonal and annual precipitations were studied at 24 stations during the period 1951–2001, using cumulative anomaly, linear trend estimation and Mann−Kendall test [26]. However, only few researches have been conducted on the comprehensive analysis of precipitation trends and characteristics of drought in time series, especially in the semi-arid Loess Plateau. Assessing the comprehensive analysis of trends and variability over the semi-arid Loess Plateau will provide the information for effective water resource management and improve the efficiency on vegetation restoration and reconstruction.

The objectives of this study are: (1) to reveal the characteristics of precipitation in 7 synoptic stations; (2) to detect the trends in monthly, seasonal, and annual time series using linear regression, Mann−Kendall tests, and Spearman’s Rho for a period of 56 years (1957–2012); (3) to consider the impact of serial correlation in detecting trends; and (4) to calculate the drought severity using SPI at the 12-month time scale in 7 synoptic stations.

## Materials and Methods

### Ethics Statement

Monthly precipitation data were collected from 7 synoptic stations (Yulin, Wuqi, Hengshan, Suide, Yan’an, Changwu, and Luochuan) from Shaanxi Province, China for the period 1957–2012. These values were obtained from the China Meteorological Data Sharing Service System (http://cdc.cma.gov.cn/cdc_en/home.dd). Therefore, this field study did not involve any endangered or protected species, and we do not require an ethics statement.

### Study site and data collection

The study area was the loess region of Northern Shaanxi which is located in the northern part of Shaanxi Province, China. The region covers an area of 149.75 km^2^. It is the most expansive portion of the loess hilly and gully region. The climate of the area is semi−arid temperate continental with a gradual transition between the four seasons of the year.

**Fig 1:**
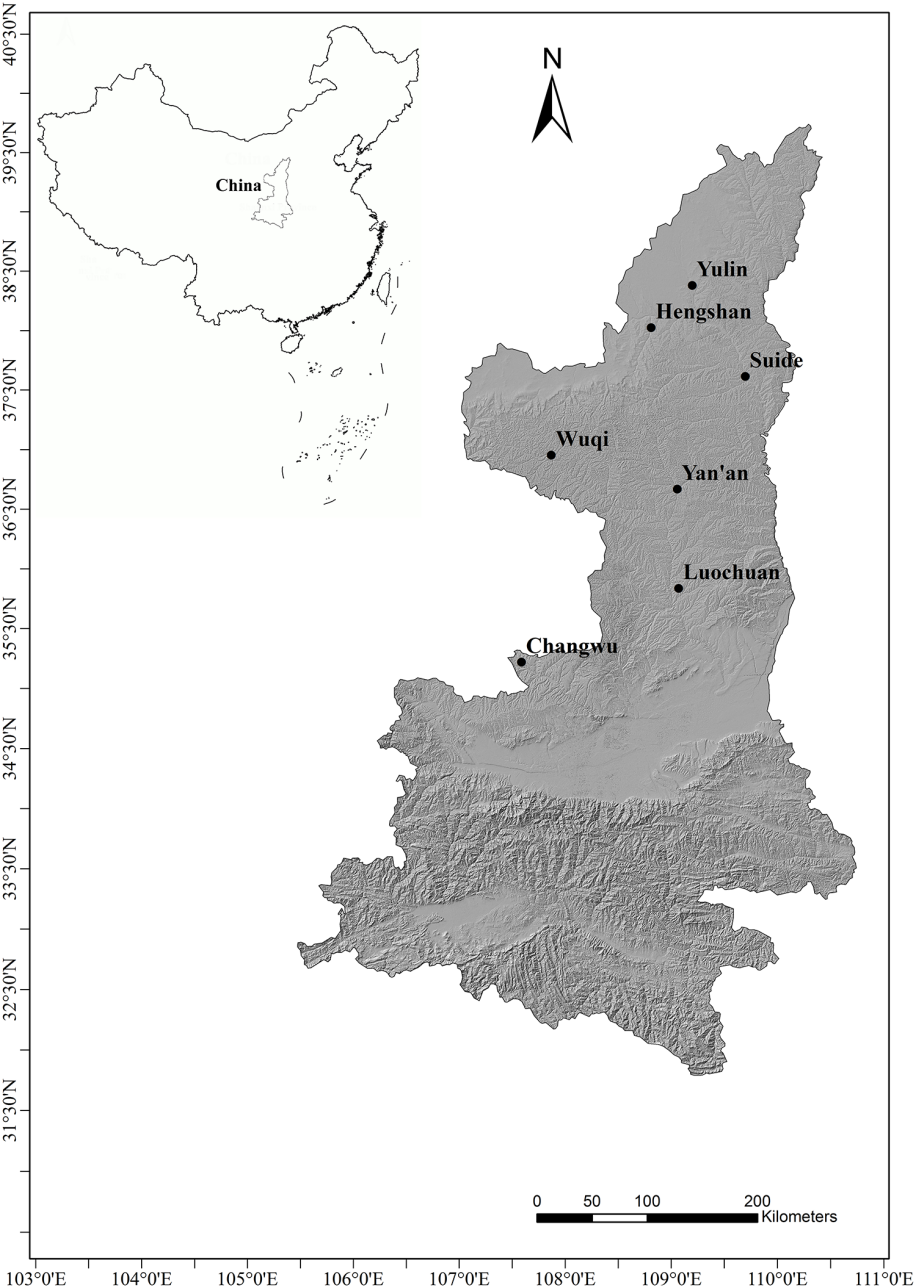
Map of the Spatial distribution of the 7 synoptic stations in the semi-arid region of Northern Shaanxi.

Monthly precipitation data were collected from 7 synoptic stations (from north to south: Yulin, Hengshan, Suide, Wuqi, Yan’an, Luochuan, and Changwu) from Shaanxi Province, China (Fig 1) during 1957–2012. These data were obtained from the China Meteorological Data Sharing Service System (http://cdc.cma.gov.cn/cdc_en/home.dd). The distribution of the 7 synoptic stations is homogeneous from north to south in the northern Shaanxi Province. A detailed description of the selected synoptic stations is given in Table 1.

**Table 1:** Geographical Descriptions of the Synoptic Stations Used in the Study.

Station No.	Name	Longitude (E)	Latitude (N)	Elevation (m a.s.l.)	Established time
53646	Yulin	109°42′	38°14′	1058.5	Jan, 1951
53738	Wuqi	108°10′	36°55′	1331.1	Oct, 1956
53740	Hengshan	109°14′	37°56′	1107.5	Jan, 1954
53754	Suide	110°13′	37°30′	928.5	Jan, 1953
53845	Yan’an	109°30′	36°36′	958.8	Jan, 1951
53929	Changwu	107°38′	35°18′	910	Sep, 1956
53942	Luochuan	109°30′	35°49′	1159.1	Nov, 1954

The semi−arid Loess Plateau is ecologically vulnerable. Agricultural production, animal husbandry, and the ecological environment rely strongly on the climatic conditions [26, 27]. Therefore, the monthly, seasonal, and annual precipitation datasets were analyzed in this study. The precipitation data were random and homogeneous. Randomness and homogeneity were tested using an autocorrelation test, Mann-Kendall trend test, and homogeneity tests of Mann-Whitney for mean and variance [28]. When the hypothesis of homogeneity failed (a significance level of 0.05), each month of the series was corrected by the method of cumulative residuals [29] using nearby homogeneous datasets as reference and assuming a confidence level of 80% [10, 30]. The quality of precipitation data was controlled with double-mass curve analysis [31].

### Study method

#### Standard Precipitation Index (SPI)

The Standard Precipitation Index (SPI) was developed to quantify the precipitation deficit for multiple time scales (1, 3, 6, 12, 24, 48 months) [32–33]. In this study, SPI is applied due to its high ability in drought identification and prediction of drought class transitions [8–10, 24–25]. The SPI value is based on the total volume of the precipitation for any given time scale and the selection of the time scale is determined according to the purposes of the study. A gamma distribution probability was adopted to describe the variation in the precipitation and an SPI value was obtained through normal standardization [25, 34]. The specific formula was described by previous studies [4, 34–35].

Table 2 displays the SPI severity drought classes [32]. We grouped the severe and extremely severe drought classes for modeling purposes since transitions referring to the extremely severe droughts are much less frequent [8].

**Table 2:** Drought classification of SPI.

Drought class	SPI value
Non-drought	SPI≥0
Mild drought	−1 < SPI < 0
Moderate drought	−1.5 < SPI≤−1
Severe/extreme drought	SPI≤−1.5

#### Mann−Kendall trend test

The Mann-Kendall test, which is also known as Kendall’s statistic, has been widely used to test for randomness in hydrology and climatology [18]. The Mann−Kendall test statistics [4, 36–37] is calculated via the following equation:

$$S\;=\;\Sigma_{i=1}^{n-1}\Sigma_{j=i+1}^{n}\text{sgn}\left(x_{j}\;-\;x_{i}\right)$$ (1)

Where *n* is the number of data points, *x*_*i*_ and *x*_*j*_ are the *i*_*th*_ and *j*_*th*_ data values in the time series (*j*>*i*), respectively, and sgn(*x*_*j*_−*x*_*i*_) is the sign function determined as:

$$\text{sgn}\left(x_{j}-x_{i}\right)\;=\{\begin{array}{ll} +1, & if\;x_{j}\;-\;x_{i}\;>\;0\\ 0, & if\;x_{j}\;-\;x_{i}\;=\;0\\ -1, & if\;x_{j}\;-\;x_{i}\;<\;0 \end{array}$$ (2)

In cases where the sample size *n*> 10, the mean (*μ*(*S*)) and variance (*σ* ^2^(*S*)) are given by the following equation:

μ(S) = 0 (3)

$$\sigma^{2}\left(S\right)\;=\;\frac{n\left(n-1\right)\left(2n+5\right)-\Sigma_{i=1}^{m}t_{i}\left(t_{i}-1\right)\left(2t_{i}+5\right)}{18}$$ (4)

where *m* is the number of tied groups and *t*_*i*_ is the number of ties of extent *i*. A tied group is a set of sample data having the same value.

In the absence of ties between the observations, the variance is calculated by the following equation:

$$\sigma^{2}\left(S\right)\;=\;\frac{n\left(n-1\right)\left(2n+5\right)}{18}$$ (5)

The standard normal test statistic *Z*_*S*_ is calculated as:

$$Z_{S}\;=\;\left\{ \begin{array}{l} \frac{S\;-\;1}{\sqrt{\sigma^{2}\left(S\right)}},if\;S\;>\;0\\ 0,if\;S\;=\;0\\ \frac{S\;+\;1}{\sqrt{\sigma^{2}\left(S\right)}},if\;S\;<\;0 \end{array}\right.$$ (6)

In this study, the significance level of *α* = 0.05 was used. If *Z*_*S*_ is a positive value, it indicates increasing trends; otherwise it represents decreasing trends. At the 5% significance level, the null hypothesis of no trend is rejected if |*Z*_*S*_| > 1.96.

#### Spearman’s Rho test

Spearman’s Rho test is a non-parametric method commonly used to verify the absence of trends. The Spearman coefficient, *D*, is the correlation coefficient of the linear regression between series *i* and *R*(*X*_*i*_), and the standardized test statistic *Z*_*D*_ is obtained from the expression [10, 38–39]:

$$D\;=\;1\;-\;\left[6\;\Sigma_{i=1}^{n}{\left(R\left(X_{i}\right)\;-\;i\right)}^{2}\right]/\left[n\left(n^{2}\;-\;1\right)\right]$$ (7)

$$Z_{D}\;=\;D\sqrt{\left(n\;-\;2\right)/\left(1\;-\;D^{2}\right)}$$ (8)

Where *n* is the number of data items in the series, *i* is the order of the elements in the original series, and R(*X*_*i*_) is the rank of *i*_*th*_ observation *X*_*i*_ in the time series.

If *Z*_*D*_ is a positive value, it indicates increasing trends; otherwise it shows decreasing trends. At the 5% significance level, the null hypothesis of no trend is rejected if |*Z*_*D*_| > 2.08.

#### Serial autocorrelation test

Temporal autocorrelation analysis correlates a time series dataset with itself at different time lags [40]. It is useful in checking randomness, locating patterns, or identifying the presence of a periodic signal in a time series dataset. For removing serial correlation from the series, the literature recommends pre-whitening the series before applying the Mann−Kendall and Spearman’s Rho tests [41]. The lag-1 serial correlation coefficient of sample data *x*_*i*_ (designated by *R*_*h*_) is expressed by [42–43]:

$$R_{h}\;=\;\frac{\frac{1}{n-1}\;\Sigma_{i=1}^{n-1}\left(x_{i}\;-\;\mu \left(x_{i}\right)\right)\cdot \left(x_{i+1}\;-\;\mu \left(x_{i}\right)\right)}{\frac{1}{n}\Sigma_{i=1}^{n}{\left(x_{i}\;-\;\mu \left(x_{i}\right)\right)}^{2}}$$ (9)

$$\mu \left(x_{i}\right)\;=\;\frac{1}{n}\;\Sigma_{i=1}^{n}x_{i}$$ (10)

Where *μ*(*x*_*i*_) is the mean of sample data and *n* is the sample size. If *R*_*h*_>0, the time series data is positive autocorrelation; if *R*_*h*_<0, the time series datais negative autocorrelation; if *R*_*h*_ = 0, the time series data shows no autocorrelation.

For the two-sided test, the *R*_*h*_ was computed by the following equation at the95% significance level[42]:

$$R_{h}\;\left(95\%\right)\;=\;\left(-1\;\pm \;1.96\;\cdot \;\sqrt{n-2}\right)\;/\;\left(n-1\right)$$ (11)

Where *n* is the sample size.

## Results and Analyses

### Characteristics of the precipitation

The basic characteristics of monthly precipitation time series at the 7 synoptic stations during 1957–2012 are summarized in Table 3. The mean monthly precipitation ranged from 31.3 to 50.7 mm. The CV of the precipitation values were very high (greater than 100%) among all the 7 stations. In addition, it appears that 2 stations in the north (Yulin and Hengshan) had the lowest mean monthly precipitation and the highest coefficients of variation (CV).

**Table 3:** Statistical parameters of monthly precipitation time series for the seven synoptic stations during 1957–2012.

Station name	Min (mm)	Max (mm)	Mean (mm)	Standard deviation (mm)	CV (%)	Skewness	Kurtosis
Yulin	0.0	330.2	33.5	45.6	136.07	2.314	6.719
Wuqi	0.0	241.2	38.7	46.3	119.72	1.641	2.488
Hengshan	0.0	208.7	31.3	39.9	127.21	1.839	3.319
Suide	0.0	307.4	37.2	46.4	124.75	1.988	4.779
Yanan	0.0	303.5	44.5	51.6	116.13	1.657	2.797
Changwu	0.0	312.0	48.3	50.8	105.11	1.704	3.541
Luochuan	0.0	293.7	50.7	54.1	106.65	1.602	2.642

CV coefficient of variation.

The time series of annual precipitation (Table 4) indicates that it had high variations at the 7 synoptic stations during 1957–2012. The highest precipitation (954.3 mm) was detected in 2003 at the Changwu station. Precipitation of 929.4 mm was detected in 2003 at the Luochuan station. The minimum precipitation (159.1 mm) was detected in 1965 at the Yulin station. Furthermore, variations of annual precipitation were larger at Yulin (CV: 26.66%) and Hengshan (CV: 25.84%) stations than the other stations (CV: ≤23.02%) based on the CV of annual precipitation.

**Table 4:** Statistical parameters of annual precipitation time series for the seven synoptic stations during 1957–2012.

Station name	Min (mm)	Max (mm)	Mean (mm)	Standard deviation (mm)	CV (%)	Skewness	Kurtosis
Yulin	159.1	692.6	402.5	107.3	26.66	0.606	0.534
Wuqi	270.0	786.3	464.0	106.8	23.02	0.640	0.783
Hengshan	165.2	686.9	376.1	97.2	25.84	0.395	0.596
Suide	254.4	745.2	446.1	101.2	22.69	0.517	0.145
Yanan	330.0	871.0	533.5	117.6	22.04	0.570	0.250
Changwu	296.0	954.3	579.6	129.3	22.31	0.517	0.222
Luochuan	341.9	929.4	608.7	125.3	20.58	0.443	0.270

### Trends analysis of precipitation

#### Serial correlation analysis

The serial correlation coefficient can improve the verification of the independence of the precipitation time series [42]. In cases where the time series are completely random, the autocorrelation function will be zero for all lags other than zero. In this study, if the value of R_h_ (95%) fell between −0.281 and 0.243 based on Eq 11 (*n* = 56), the null hypothesis H_0_: R_h_ = 0 (that the time series data shows no autocorrelation) was accepted [4]. The precipitation time series data can be analyzed by Mann−Kendall test and the Spearman’s Rho test only if the null hypothesis H_0_ was accepted.

Table 5 shows lag-1 serial correlation coefficients. The serial correlations coefficients ranged from −0.280 to −0.053 at the annual scale, and the serial correlations coefficients ranged from −0.269 to −0.193 at the seasonal scale. Therefore, they fell between−0.281 and 0.243, and the null hypothesis was accepted. The existence of the serial correlation had scarcely any effects on the Mann−Kendall and Spearman’s Rho tests.

**Table 5:** Lag–1 serial correlation coefficients for precipitation data.

Station name	Spring	Summer	Autumn	Winter	annual
Yulin	0.171	−0.029	−0.009	0.019	−0.064
Wuqi	0.027	−0.227	0.037	−0.018	−0.239
Hengshan	0.137	−0.079	0.052	0.026	−0.055
Suide	0.085	0.033	−0.113	−0.090	−0.053
Yanan	−0.027	−0.122	0.066	−0.018	−0.189
Changwu	0.034	−0.269	0.049	0.193	−0.238
Luochuan	0.097	−0.269	0.079	−0.024	−0.280

#### Trends analysis of different time scale of precipitation series

The Mann−Kendall test, Spearman’s Rho test, and linear regression were applied to analyze trends of precipitation at the 5% significance level. The results of these tests are presented in Table 6.

**Table 6:** Results of the statistical tests for monthly precipitation during 1957–2012.

		Month											
Station	Test	Jan.	Feb.	Mar.	Apr.	May	Jun.	Jul.	Aug.	Sep.	Oct.	Nov.	Dec.
Yulin	Z_S_	–0.536	0.602	–0.127	–1.392	0.664	0.848	–2.050	–0.565	0.113	–0.700	–0.999	0.410
Yulin	Z_D_	–0.553	0.582	–0.132	–1.399	0.761	0.896	–1.979	–0.464	0.110	–0.686	–0.843	0.434
Yulin	B(mm/year)	–0.010	0.013	–0.007	–0.239	0.096	0.348	–0.673	–0.257	0.043	–0.090	–0.021	0.012
Wuqi	Z_S_	–0.775	0.467	–0.099	–1.894	–0.488	1.018	–0.417	–0.714	–0.495	–0.686	–0.998	0.866
Wuqi	Z_D_	–0.798	0.287	–0.044	–1.946	–0.486	1.100	–0.538	–0.575	–0.523	–0.694	–0.903	0.941
Wuqi	B(mm/year)	–0.002	0.023	–0.001	–0.283	–0.189	0.320	–0.380	–0.318	–0.228	–0.095	–0.057	0.007
Hengshan	Z_S_	0.206	0.983	0.035	–1.675	0.269	1.802	–1.838	–1.513	0.247	–0.594	–0.716	0.709
Hengshan	Z_D_	0.184	0.896	0.110	–1.745	0.316	1.881	–1.777	–1.523	0.088	–0.649	–0.568	0.768
Hengshan	B(mm/year)	0.009	0.030	0.038	–0.259	0.024	0.531	–0.634	–0.772	0.039	–0.104	–0.091	0.014
Suide	Z_S_	–0.637	0.184	–0.629	–1.548	–0.940	–0.792	–1.364	–0.742	–0.262	–0.679	–1.174	–0.549
Suide	Z_D_	–0.716	0.191	–0.634	–1.453	–0.888	–0.888	–1.461	–0.746	–0.279	–0.671	–1.115	–0.575
Suide	B(mm/year)	–0.001	0.026	–0.024	–0.225	–0.248	–0.098	–0.579	–0.510	–0.155	–0.196	–0.027	–0.002
Yan’an	Z_S_	–0.113	1.040	–0.636	–1.336	–0.558	0.502	–1.505	–0.954	–0.869	–0.014	–1.132	–0.488
Yan’an	Z_D_	–0.110	1.001	–0.642	–1.368	–0.575	0.486	–1.469	–0.956	–0.806	–0.015	–1.145	–0.479
Yan’an	B(mm/year)	–0.004	0.019	–0.043	–0.210	–0.175	0.129	–0.795	–0.476	–0.442	–0.053	–0.080	–0.008
Changwu	Z_S_	1.189	1.597	–0.912	**−3.060** *	–0.650	0.954	0.516	0.261	0.368	–0.940	**−2.142** *	1.056
Changwu	Z_D_	1.115	1.634	–1.001	**−3.131** *	–0.530	0.956	0.560	0.279	0.390	–0.941	**−2.085** *	1.115
Changwu	B(mm/year)	0.079	0.101	–0.069	**−0.488**	–0.213	0.383	0.069	0.233	0.011	–0.317	**−0.249**	0.029
Luochuan	Z_S_	0.106	1.301	–1.428	**−2.509** *	–0.127	1.491	0.000	–0.283	0.304	–0.028	–1.520	0.341
Luochuan	Z_D_	0.037	1.322	–1.578	**−2.543** *	–0.044	1.252	0.000	–0.198	0.221	–0.051	–1.634	0.449
Luochuan	B(mm/year)	0.052	0.081	–0.142	**−0.457**	–0.098	0.451	0.101	–0.061	0.116	–0.070	–0.196	0.025

Z_S_ Mann–Kendall test;Z_D_ Spearman’s Rho test; b Slope of linear regression. Bold characters represent trends identified by 2 statistical methods.

* Statistically significant trends at the 5% significance level.

**Fig 2:**
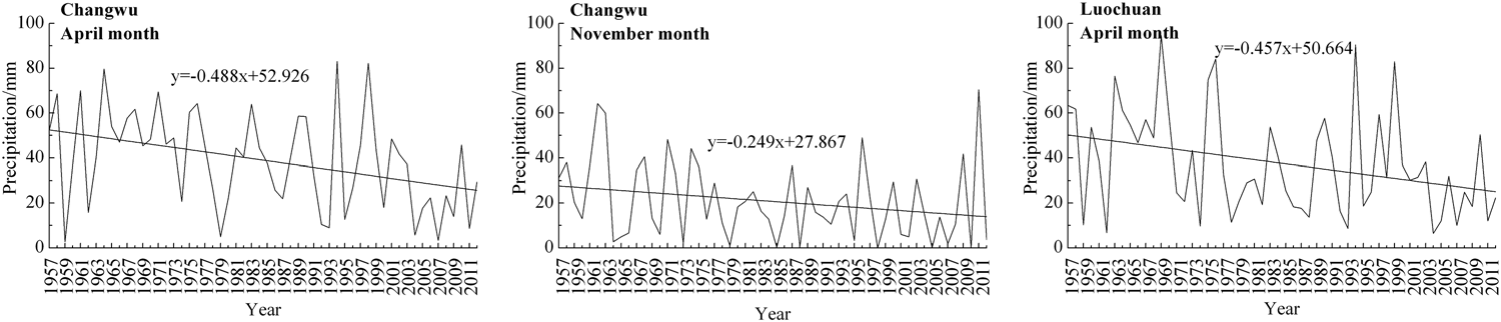
Variations of monthly precipitation at the stations with significant trends during the study period.

The statistical tests for the monthly precipitation series during 1957 −2012 (Table 6) indicated at the Changwu station had a significant decreasing trend in April and November with change rates of −0.488 mm/year and −0.249 mm/year, respectively. Meanwhile, a similar significant decreasing trend was found in April with a slope of −0.457 mm/year at the Luochuan station. The significant increasing trend was not detected at all the stations. The magnitudes of the significant trends in the monthly precipitation series for the stations mentioned above are presented in Fig 2.

The results (Table 6) also suggest that all stations exhibited an increasing trend in February and decreasing trends in April, October, and November. However, both increasing and decreasing trends were obtained in the other months.

On the seasonal scale (Table 7), decreasing trends for precipitation were found only in the spring for all the stations because of the negative *Z*_*S*_ and *Z*_*D*_. However, there were the significant increasing trends in winter with the slope of 0.220 mm/year and significant decreasing trends in spring with the slope of −0.770 mm/year only at the Changwu station.

**Table 7:** Results of the statistical tests for seasonal and annual precipitation during 1957–2012.

		Season				
Station	Test	Spring	Summer	Autumn	Winter	Annual
Yulin	Z_S_	−0.205	−0.735	−0.021	0.021	−0.714
Yulin	Z_D_	−0.272	−0.791	−0.110	0.162	−0.664
Yulin	B(mm/year)	−0.137	−0.582	−0.112	0.017	−0.816
Wuqi	Z_S_	−1.604	−0.558	−0.615	0.134	−0.968
Wuqi	Z_D_	−1.626	−0.471	−0.634	0.154	−0.986
Wuqi	B(mm/year)	−0.472	−0.378	−0.380	0.014	−1.248
Hengshan	Z_S_	−0.353	−1.315	0.417	1.138	−1.272
Hengshan	Z_D_	−0.397	−1.414	0.147	1.077	−1.183
Hengshan	B(mm/year)	−0.197	−0.875	0.052	0.054	−1.071
Suide	Z_S_	−1.138	−1.788	−0.544	−0.141	**−2.460** *
Suide	Z_D_	−1.337	−1.857	−0.479	−0.206	**−2.430** *
Suide	B(mm/year)	−0.496	−1.186	−0.324	−0.019	**−2.034**
Yan’an	Z_S_	−0.940	−1.611	−0.954	0.834	**−2.021** *
Yan’an	Z_D_	−1.016	−1.610	−0.858	0.791	**−2.193** *
Yan’an	B(mm/year)	−0.427	−1.142	−0.575	0.018	**−2.129**
Changwu	Z_S_	**−2.276** *	0.784	−0.919	**2.672** *	−0.417
Changwu	Z_D_	**−2.277** *	0.881	−0.806	**2.764** *	−0.346
Changwu	B(mm/year)	**−0.770**	0.684	−0.577	**0.220**	−0.453
Luochuan	Z_S_	−1.908	0.198	−0.254	1.470	−0.580
Luochuan	Z_D_	−1.849	0.125	−0.257	1.484	−0.456
Luochuan	B(mm/year)	−0.697	0.289	−0.381	0.162	−0.633

Z_S_ Mann–Kendall test;Z_D_ Spearman’s Rho test; b Slope of linear regression. Bold characters represent trends identified by 2 statistical methods.

* Statistically significant trends at the 5% significance level.

All stations exhibited a decreasing trend in annual precipitation series (Table 7). However, significant decreasing trends in annual precipitation were detected at Suide and Yan’an stations with the slope of −2.034 mm/year and– 2.129 mm/year, respectively, the other stations had no significant trends.

### Analysis of SPI

#### Analysis of different time scales of SPI

**Fig 3:**
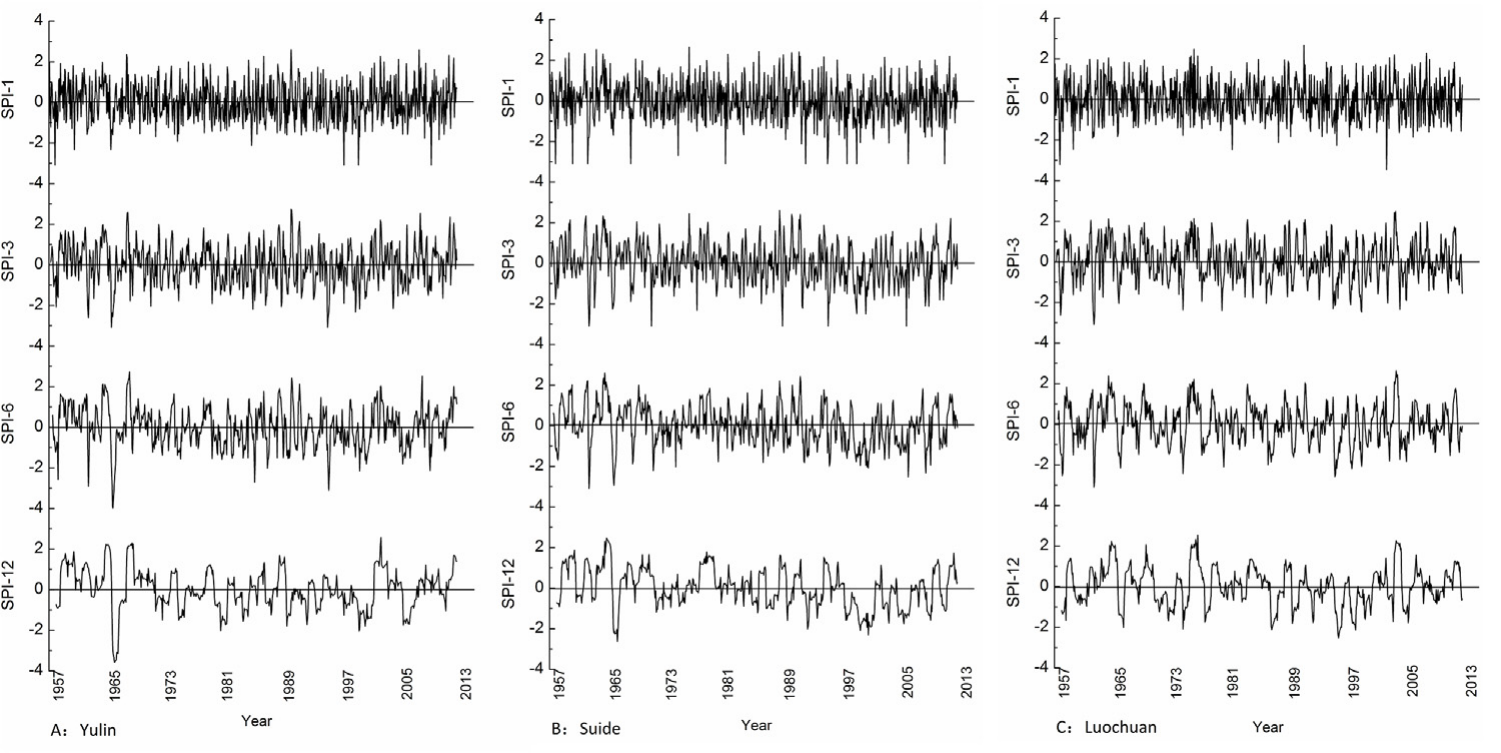
Different time scales of SPI (SPI-1, SPI-3, SPI-6, SPI-12) at 3 representative synoptic stations from north to south during 1957–2012.

Fig 3 shows the SPI (SPI-1, SPI-3, SPI-6, SPI-12) of different time scales at the Yulin, Suide and Luochuan synoptic stations (3 representative synoptic stations from north to south) during 1957–2012. The SPI on shorter time scales (say 1 and 3 months) was frequent fluctuations around the horizontal axis and affected by short time precipitation. While on the longer time scales (6 and 12 months), the responses of SPI-6 and SPI-12 on short time precipitation change into tardiness. The periodicity of drought and flood change is relatively stable.

For dry climate where precipitation is seasonal in nature and zero values are common, there will be too many zero precipitation values in a particular season. In the dry climatic zones, the calculated SPI values at short time scales may not be normally distributed because of the highly skewed underlying precipitation distribution and limitation of fitted gamma distribution. This may be leading to large errors while simulating precipitation distributions in dry climates from small data samples [12]. Therefore, in this study, we analyzed the SPI at a 12-month time scale for managing water resources of a certain region and identifying the persistence of dry periods.

#### Analysis of drought characteristics

The characteristics of droughts at the 12-month time scale are presented in Table 8. It should be noted that the most severe drought for 3 stations (Yulin, Hengshan, and Suide) occurred in 1965. The most severe drought year was 1995 at the Changwu and Luochuan stations and 1986 at the Wuqi station. However, the most severe drought year was 1974 at the Yan’an station.

**Table 8:** Characteristics of droughts at 12-month time scale.

	The most severe drought		Percentage of drought years during the observed period		
Station name	SPI	Year	Moderate (%)	Severe/extreme (%)	Total(%)
Yulin	−3.57	1965	9	4	13
Wuqi	−2.14	1986	9	7	16
Hengshan	−2.37	1965	18	4	22
Suide	−2.27	1965	7	6	13
Yan’an	−2.01	1974	5	9	14
Changwu	−3.01	1995	9	4	13
Luochuan	−2.52	1995	11	5	16

The percentage of drought years during the observed period at the stations is presented in Table 8. The total percentage of drought years ranged from 13% (at Yulin, Suide and Changwu stations) to 22% (at Hengshan station). However, the percentage of severe/extreme drought years was from 4% (at Yulin, Hengshan and Changwu stations) to 9% (at Yan’an station). The percentage of moderate drought years was from 5% (at Yan’anstation) to 18% (at Hengshanstation). Therefore, the drought degree of Yulin and Changwu was slightly less than that of Suide. During 1957–2012, the drought degree of Yulin and Changwu was the lowest and that of Hengshan was the highest among the 7 synoptic stations.

## Conclusions and Discussion

In the semi−arid Loess Plateau, precipitation plays an essential role in ecological restoration and reconstruction. In this study, a complete picture of precipitation is presented during 1957–2012 over the northern part of Shaanxi Province, China.

The fluctuation of monthly precipitation was intense (coefficients of variation >100%) at all the stations. The main reason was that fractured topography of the North Shaanxi Loess Plateau formed into microenvironment. Meanwhile, the effects of monsoon and broken topography on monthly precipitation were found by Wang [14], Liu [20] and Sun [44] in the Yellow River Basin, in the eastern region of Gansu and in the Loess Plateau area of Shaanxi of China, respectively. The variations of annual precipitation were prominent at Yulin and Hengshan stations than the other stations based on the CV of annual precipitation. Because Yulin and Hengshan stations located on the border between the North Shaanxi Loess Plateau and the Maowusu Desert, their climate was drought [44].

The significantly decreasing or increasing trend of precipitation was displayed at different time scale owing to climate change and difference of regional geographical environment [26]. Climate change was affected by Qinling Mountain, winter monsoon and summer monsoon. The Qinling Mountain had hindrance function for the movement of winter monsoon and summer monsoon. However, the difference of regional geographical environment resulted by microtopography types existed in the northern Shaanxi. In addition, these regional differences may be due to the spatial distribution of precipitation and NDVI differences [14, 19, 44–45]. Meanwhile, Sun et al. [44] found that the drought strength reduced by different rates in summer, autumn, and winter. However, the drought strength has increased in spring since 2001 in the Loess Plateau area of Shaanxi, China. These results are similar to those of this study.

However, the SPI−12 series analysis suggests that the drought degree of Yulin and Changwu was the lowest and that of Hengshan was the highest among the 7 synoptic stations. Hengshan station is located in the south edge of Maowusu Desert, where vegetation was scarce and the drought trend was much more obvious [26]. The results of SPI−12 series were similar to results of annual precipitation series analysis, and were similar to previous results [19, 20, 45].

The results of precipitation and SPI-12 series can be helpful for planning the efficient use of water resources for hydroelectric and agricultural production. Further research on analyzing the spatial variation of precipitation trends, relationships with climate change, and the relation between precipitation and NDVI [20, 45] are recommended. Moreover, future work should be oriented towards developing an information system for monitoring and early drought prediction.
